# Short-echo-time magnitude image derived from quantitative susceptibility mapping could resemble neuromelanin-sensitive MRI image in substantia nigra

**DOI:** 10.1186/s12883-020-01828-8

**Published:** 2020-06-30

**Authors:** Xue Ling Liu, Li Qin Yang, Feng Tao Liu, Pu-Yeh Wu, Yong Zhang, Han Zhuang, Yong Hong Shi, Jian Wang, Dao Ying Geng, Yu Xin Li

**Affiliations:** 1 Department of Radiology, Huashan Hospital, Fudan University, Shanghai, 200040 China; 2 Institute of Functional and Molecular Medical Imaging, Fudan University, Shanghai, 200040 China; 3 Department of Neurology, Huashan Hospital, Fudan University, Shanghai, 200040 China; 4 GE Healthcare China, Beijing, 100176 China; 5 Shanghai Key Laboratory of Medical Imaging Computing and Computer-Assisted Intervention, School of Basic Medical Sciences, Fudan University, Shanghai, 200032 China

**Keywords:** Parkinson disease, Substantia nigra, Pars compacta, Quantitative susceptibility mapping, Neuroimaging

## Abstract

**Background:**

In this study, we explored whether the proposed short-echo-time magnitude (setMag) image derived from quantitative susceptibility mapping (QSM) could resemble NM-MRI image in substantia nigra (SN), by quantitatively comparing the spatial similarity and diagnosis performances for Parkinson’s disease (PD).

**Methods:**

QSM and NM-MRI were performed in 18 PD patients and 15 healthy controls (HCs). The setMag images were calculated using the short-echo-time magnitude images. Bilateral hyperintensity areas of SN (SN_hyper_) were manually segmented on setMag and NM-MRI images by two raters in a blinded manner. The inter-rater reliability was evaluated by the intraclass correlation coefficients (ICC) and the Dice similarity coefficient (DSC). Then the inter-modality (i.e. setMag and NM-MRI) spatial similarity was quantitatively assessed using DSC and volume of the consensual voxels identified by both of two raters. The performances of mean SN_hyper_ volume for PD diagnosis on setMag and NM-MRI images were evaluated using receiver operating characteristic (ROC) analysis.

**Results:**

The SN_hyper_ segmented by two raters showed substantial to excellent inter-rater reliability for both setMag and NM-MRI images. The DSCs of SN_hyper_ between setMag and NM-MRI images showed substantial to excellent voxel-wise overlap in HCs (0.80 ~ 0.83) and PD (0.73 ~ 0.76), and no significant difference was found between the SN_hyper_ volumes of setMag and NM-MRI images in either HCs or PD (p > 0.05). The mean SN_hyper_ volume was significantly decreased in PD patients in comparison with HCs on both setMag images (77.61 mm^3^ vs 95.99 mm^3^, *p* < 0.0001) and NM-MRI images (79.06 mm^3^ vs 96.00 mm^3^, *p* < 0.0001). Areas under the curve (AUCs) of mean SN_hyper_ volume for PD diagnosis were 0.904 on setMag and 0.906 on NM-MRI images. No significant difference was found between the two curves (*p* = 0.96).

**Conclusions:**

SN_hyper_ on setMag derived from QSM demonstrated substantial spatial overlap with that on NM-MRI and provided comparable PD diagnostic performance, providing a new QSM-based multi-contrast imaging strategy for future PD studies.

## Background

Parkinson’s disease (PD) is characterized by the progressive loss of neuromelanin-containing dopaminergic neurons [1] in the substantia nigra pars compacta (SNc). Neuromelanin-sensitive MRI (NM-MRI) technique [1, 2] is currently used for imaging SNc with hyperintensity, which has been shown positively related to the quantity of neuromelanin-containing neurons by postmortem study [3]. Another pathological hallmark in PD is iron deposition throughout SN [4]. T2*-weighted imaging based on the paramagnetic magnetic susceptibility of iron, such as susceptibility-weighted imaging (SWI) and quantitative susceptibility mapping (QSM), are commonly used to reveal the iron deposition in PD [5, 6].

Previous evidence suggests the roles of neuromelanin and iron are intricate and it is well-recognized that iron deposition is related to the reduction of neuromelanin in SNc in PD [4]. Several studies [7, 8] in PD have conducted NM-MRI and T2*-weighted imaging, individually, to assess the neurodegenerative changes in SNc. However, the two-sequence approaches are time consuming and inter-modality registration is needed. If there is a neuroimaging technique with multi-contrast both sensitive to neuromelanin and iron, it could be valuable for the study of pathogenesis associated with PD.

In 2015, Langley et al. [9] proposed a technique with two echoes using GRE sequence to generate two contrasts, the first echo taken as the images sensitive to neuromelanin in SNc and the second echo used to generate the susceptibility images sensitive to iron. As we known, QSM is a reliable quantitative technique for magnetic susceptibility assessment [10]. Based on the magnitude and phase images generated from GRE complex data, susceptibility images could be calculated and used for quantitatively assessment of the iron deposition in PD [5, 6]. However, up to now, no study about neuromelanin-sensitive contrast in SNc using QSM has been reported. Empirically, we noticed that there was a pair of hyperintensity crescent-shaped structures in the midbrain on the first several short-echo-time magnitude images from QSM, which resemble the previously reported appearance of SNc on NM-MRI images.

In this study, we proposed a new parameter image based on the short-echo-time magnitude images from QSM, denoted as setMag. We hypothesized that the midbrain hyperintensity areas on setMag and SNc on NM-MRI images are spatially congruent, and that the morphology changes on setMag images could have comparable diagnostic performance as NM-MRI images in PD. Therefore, we quantitatively evaluated the spatial overlap of hyperintensity area in the midbrain on setMag images and SNc on NM-MRI images, and compared their diagnostic performances in PD, aiming to demonstrate the values of setMag for providing neuromelanin-sensitive contrast and serving as a candidate imaging biomarker in future PD studies.

## Methods

### Subjects

Eighteen PD patients and age-matched 15 healthy controls (HCs) were recruited in this study. Patients with PD were diagnosed by two experts of extrapyramidal motor disease in the neurology department (Feng Tao Liu and Jian Wang), according to the movement disorder society (MDS) clinical diagnostic criteria for PD [11]. All healthy controls were recruited from the community with no history of neuropsychiatric or neurological diseases. Exclusion criteria were as follows: no history of other neurological/psychiatric disorders including Parkinson-plus syndrome, substance abuse, severe infection, and tremor-related dysmetabolism including thyroid dysfunction and drug toxicity.

The motor dysfunction of PD subjects was further evaluated using the Movement Disorder Society Unified Parkinson’s Disease Rating Scale Part III [12] (MDS-UPDRS Part III) and Hoehn and Yahr scale [13] in the OFF medication state. Disease duration was calculated as the time between the onset of motor symptoms as reported by subjects and the time for MRI scan. This study was approved by the Ethics Committee of Huashan Hospital, Fudan University (approval No. KY 2016–214). Written consent was obtained from each participant.

### Image acquisition

All MR examinations were performed on a 3.0 T MR750 scanner (GE Healthcare, Milwaukee, WI) equipped with an eight-channel head matrix coil at the Department of Radiology of Huashan Hospital of Fudan University, China. Foam padding was applied to prevent head movement for each participant, and earplugs were provided to reduce scanner noise.

The T1-weighted FSE NM-MRI sequence was as per Sasaki et al. [1], and parameters were as follows: repetition time/echo time (TR/TE) = 600/13 ms, bandwidth = 31.25 kHz, flip angle = 145°, field of view (FOV) = 240 × 240 mm, matrix size = 512 × 320, slice thickness = 1.5 mm, voxel size = 0.47 × 0.75 × 1.5 mm^3^, number of slices = 16, NEX = 5, acquisition time = 8:03 min. The 3D multi-gradient-echo QSM sequence parameters were as follows: TR = 41.6 ms, number of echoes = 16, first TE = 3.2 ms, TE spacing = 2.4 ms, bandwidth = 62.50 kHz, flip angle = 12°, FOV = 256 × 256 mm, matrix size = 256 × 256, slice thickness = 1 mm, voxel size = 1 × 1 × 1 mm^3^, number of slices = 140, acceleration factor = 2, acquisition time = 9:00 min. In addition, in order to exclude other pathological diseases in the mesencephalon, conventional MRI scans including T2-weighted fluid-attenuated inversion recovery (FLAIR) and diffusion-weighted images (DWI) were also acquired prior to the NM-MRI and QSM sequences. All sequences were scanned using axial sections parallel to the anterior commissure-posterior commissure (AC-PC) line, with whole-brain coverage for QSM and the two routine MRI sequences, and with coverage from the upper margin of the mammillary body to the pons for NM-MRI.

### Image preprocessing and SN segmentation

A total of 16 magnitude images (denoted as Mag_i_, where i represent number of 1–16) were generated by QSM data for each subject. The first magnitude image, corresponding to the shortest TE, has minimal T2*-weighted contrast and significant T1-weighted contrast especially around SN. As the echo time increases, the contribution of T2*-weighted contrast increases gradually [14]. Hypointensity in iron-riched red nucleus (RN), which may represent the emerging of T2* contrast, was shown from Mag_4_ to Mag_16_ in our data. Thus, a calculated short-echo-time magnitude image with strengthened T1-weighted contrast, denoted as setMag, was defined using the following formula:

$$ \mathrm{setMag}={\left(\sqrt{\sum \left({Mag_i}^2\right)}\right)}^4 $$

where i = 1/2/3, corresponding to shortest three TEs (3.2/5.6/8.0 ms) in this study. The NM-MRI images were co-registered to the setMag images before segmentation. Preprocessing was done using MATLAB (MathWorks, Natick, MA) and Statistical Parametric Mapping (SPM12) (http://www.fil.ion.ucl.ac.uk/spm/).

**Fig. 1:**
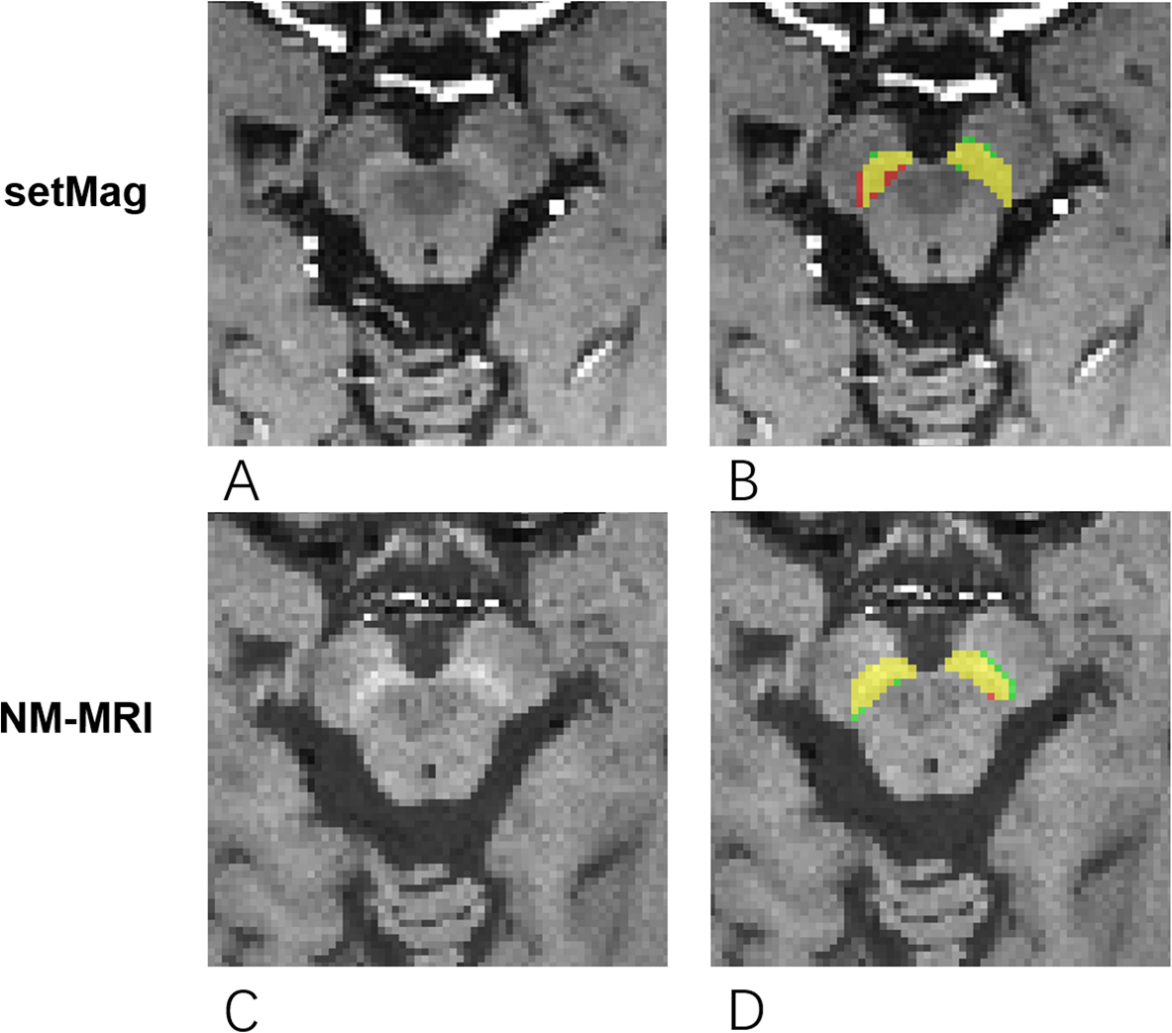
The segmentation of SN_hyper_ regions of interest (ROI) on setMag and NM-MRI images of a representative subject. A zoomed in view of midbrain area on the setMag and NM-MRI images are shown in **a** and **c**, respectively. The corresponding left and right SN_hyper_ regions of interest (ROI) segmented by rater 1 (red), rater 2 (green) and their consensual voxels (yellow) are shown in **b** and **d**

Hyperintensity areas of substantia nigra (SN_hyper_) were manually segmented on setMag and NM-MRI images in a blinded manner using ITK-SNAP software [15] by two radiologists with more than five (rater 1, Xue Ling Liu) and 10 (rater 2, Yu Xin Li) years of experience in neuroimaging, respectively. For each subject, left and right SN_hyper_ regions of interest (ROIs) were respectively segmented in three consecutive axial slices that internal to cerebral peduncle with best contrast around this region (Fig. 1).

### Inter-rater and inter-modality quantitative spatial similarity analysis

The spatial overlap of each pair of ROIs segmented either by two different raters (i.e. inter-rater) or on two different modalities (setMag and NM-MRI) (i.e. inter-modality) was compared using the Dice similarity coefficient (DSC) [16], and was defined as

$$ \mathrm{DSC}=\frac{2\times V\ \left(A\cap B\right)}{V\ (A)+V\ (B)} $$

V is the total volume of left or right ROI. Letters A and B denotes ROIs from different raters or different modalities. The operator ∩ represents the intersection area.

### Evaluation of diagnostic performance in PD

The performance of mean volume of left and right SN_hyper_ derived from setMag images in differentiating PD from HCs were conducted using receiver operating characteristic (ROC) analysis, and was compared with that of NM-MRI images with Medcalc software [17]. The significance level was set at *P* < 0.05 (two-tailed).

### Statistical analysis

For comparison of gender between PD and HC groups, chi-square test was used. For age and mean SN_hyper_ volume between two groups, data were first tested for normality with the D’Agostino-Pearson omnibus Normality test, then compared using independent samples *t*-tests or Mann-Whitney test. For inter-modality comparison between setMag and NM-MRI images, paired *t*-test or Wilcoxon test were performed. The statistical agreement of SN_hyper_ volume between two rates was assessed using the intraclass correlation coefficients (ICC) with a two-way random method, and determined using the following criteria: (0.8, 1] = excellent agreement and (0.6, 0.8] = substantial agreement [18]. The correlations between mean SN_hyper_ volume and disease severity (UPDRSIII score and H-Y stage) were used by Spearman correlation analysis. The above statistical analyses were performed using GraphPad Prism Software version 7.0 (GraphPad Prism Software Inc., San Diego, CA).

## Results

### Demographic and clinical information

Demographic and clinical information from HCs and patients with PD are shown in Table 1. There were no significant differences in gender (*p* = 0.23) nor age (*p* = 0.09) between the two groups. Disease duration for PD subjects ranged from 4 to 59 months (21.22 ± 14.60 months), UPDRS-III scores ranged from 4 to 60 (21.78 ± 13.96) and H&Y score ranged from 1 to 3 (H&Y 1: *n* = 10, H&Y 2: *n* = 4 and H&Y 3: *n* = 4).

**Table 1:** Demographic information and clinical characteristics of healthy controls and PD patients

Variable	HCs (*n* = 15)	PD (*n* = 18)	*p*
Gender (male: female)	9:06	7:11	0.23
Age (median (range), year)	58 (43–66)	61 (40–79)	0.09
Disease duration (month)	–	21.22 ± 14.60	–
MDS UPDRS-III score	–	21.78 ± 13.96	–
H-Y stage (median (range))	–	1 (1–3)	–

### Inter-rater reliability on setMag and NM-MRI images

Table 2 shows the inter-rater reliability of the SN_hyper_ segmented by two raters, quantitated by the ICC of the SN_hyper_ volume and the DSC of the SN_hyper_ ROIs, for setMag or NM-MRI images respectively. All values revealed substantial to excellent inter-rater reliability. Specifically, the ICCs of the SN_hyper_ volume were ranged from 0.72 to 0.91 in HCs, and from 0.70 to 0.94 in PD, respectively, and the DSCs of the SN_hyper_ ROI were ranged from 0.89 to 0.93 in HCs, and from 0.86 to 0.90 in PD, respectively.

**Table 2:** Inter-rater reliability on setMag and NM-MRI images

	ICCs of the SN_hyper_ volume				DSCs of the SN_hyper_ ROI			
	setMag		NM-MRI		setMag		NM-MRI	
	Left	Right	Left	Right	Left	Right	Left	Right
HCs	0.80	0.91	0.77	0.72	0.92 ± 0.02	0.93 ± 0.01	0.89 ± 0.06	0.93 ± 0.04
PD	0.77	0.93	0.70	0.94	0.89 ± 0.08	0.89 ± 0.08	0.86 ± 0.07	0.90 ± 0.05

For following analysis, only the consensual voxels (i.e. the voxels identified by both of two raters) were selected as the left and right SN_hyper_ ROIs of setMag and NM-MRI images for each subject.

### SN_hyper_ similarities between setMag and NM-MRI images

SN_hyper_ similarities between setMag and NM-MRI images were estimated through both DSC and volume. As shown in Table 3, the left, right and mean SN_hyper_ ROIs between setMag and NM-MRI images have substantial to excellent spatial overlap in either HC or PD group, with DSCs of 0.80 ± 0.05 and 0.83 ± 0.04 for left and right SN_hyper_ in HCs, and 0.76 ± 0.09 and 0.73 ± 0.07 for left and right SN_hyper_ in PD, respectively. Besides, there were no significant differences between the mean SN_hyper_ volumes of setMag and NM-MRI images in either HCs (*p* = 0.45 for left, and *p* = 0.31 for right) or PD (*p* = 0.36 for left, and *p* = 0.053 for right) group.

**Table 3:** The DSC and volume of consensual voxels identified by both of two raters

Group			Left	Right	Mean
HCs	DSC		0.80 ± 0.05	0.83 ± 0.04	0.81 ± 0.04
	Volume	setMag	97.67 ± 11.34	94.33 ± 12.61	95.99 ± 10.60
		NM-MRI	94.53 ± 11.11	97.47 ± 9.32	96.00 ± 8.49
		p	0.45	0.31	0.99
PD	DSC		0.76 ± 0.093	0.73 ± 0.067	0.74 ± 0.07
	Volume	setMag	83.28 ± 14.54	71.94 ± 16.73	77.61 ± 13.06
		NM-MRI	79.78 ± 13.45	78.33 ± 15.99	79.06 ± 11.93
		p	0.36	0.053	0.62

p represents the comparison of the volume between SN_hyper_ of setMag and NM-MRI images using paired t-test or Wilcoxon test

### Diagnostic performance in PD

**Fig. 2:**
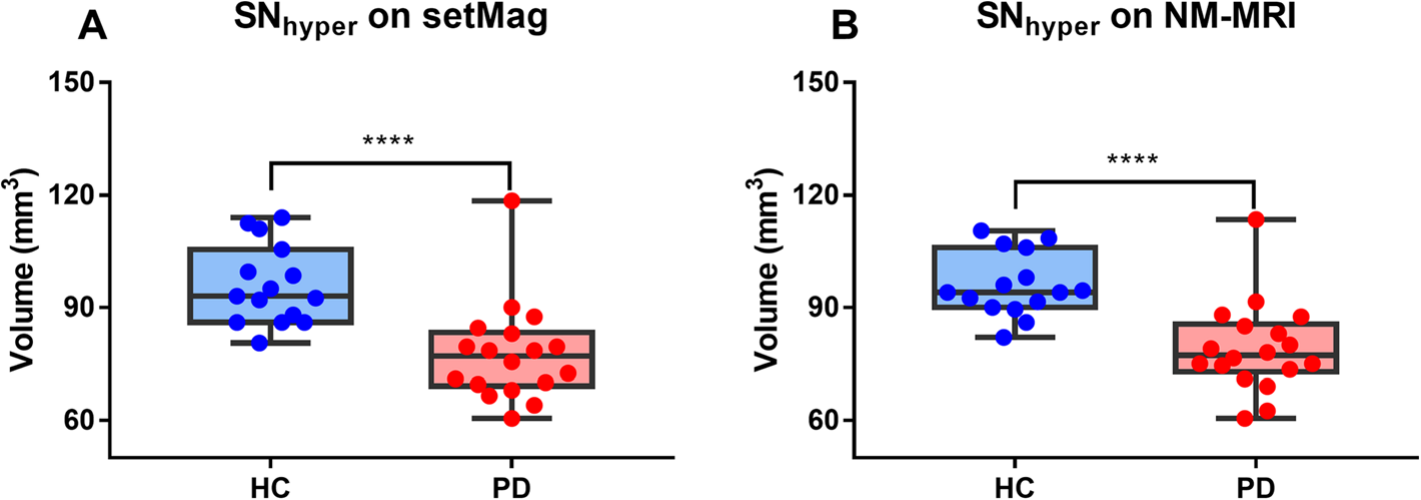
Comparison of the mean SN_hyper_ volume on setMag and NM-MRI images. Comparison of the mean SN_hyper_ volume on both setMag (A) and NM-MRI (B) images for HCs and PD patients. The scatter-box diagram denotes the 25th and 75th percentiles with the line denoting the mean value. Significant differences between PD and HCs are represented as: ****P < 0.0001

The mean SN_hyper_ volume was significantly decreased in PD patients compared with HC group on both setMag images (77.61 ± 13.06 mm^3^ vs 95.99 ± 10.60 mm^3^, *p* < 0.0001) and NM-MRI images (79.06 ± 11.93 mm^3^ vs 96.00 ± 8.49 mm^3^, *p* < 0.0001) (Fig. 2).

**Fig. 3:**
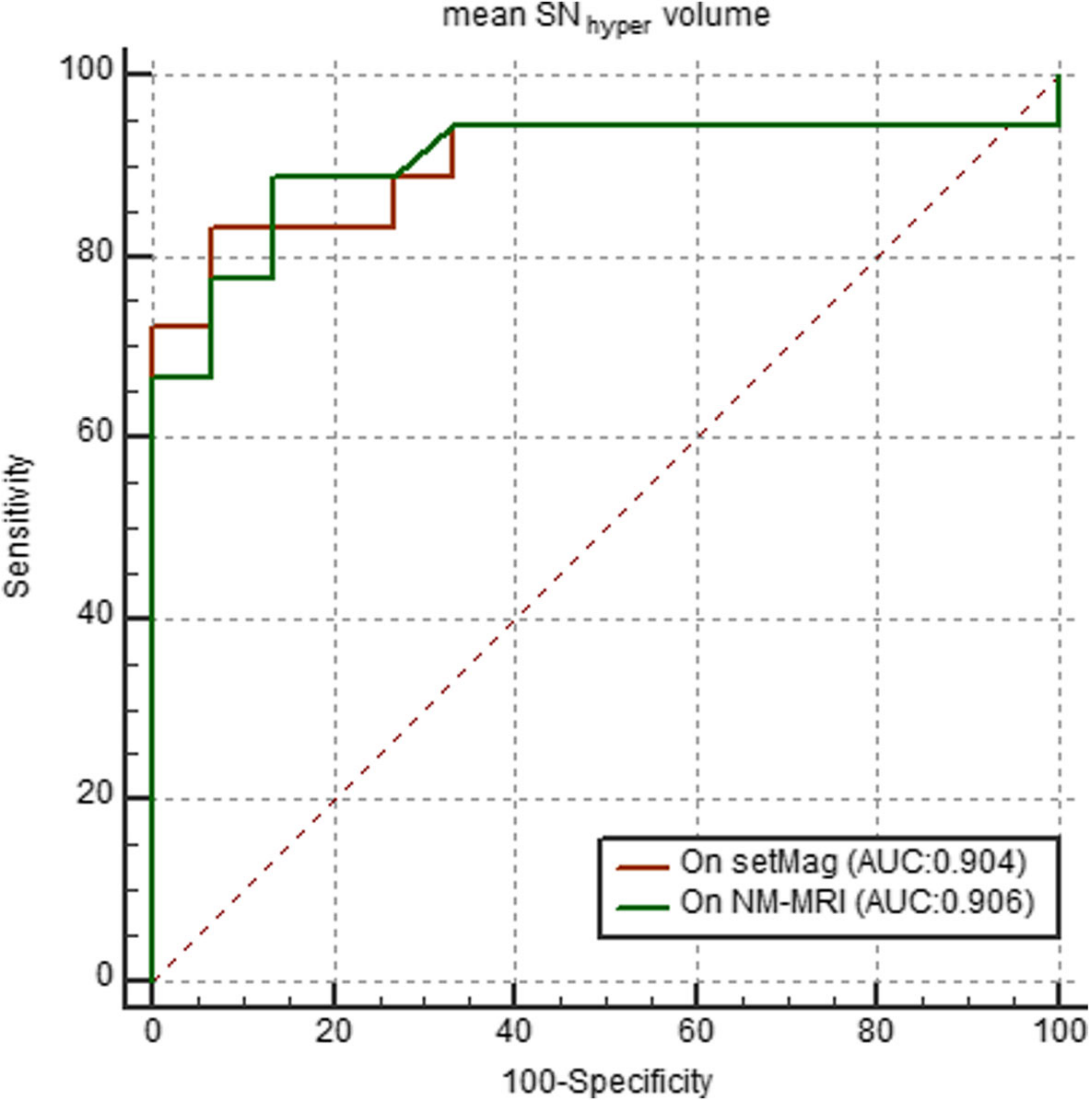
Receiver operator characteristic analyses of the mean SN_hyper_ volume for differentiating PD from HCs on setMag and NM-MRI images. There was no significant difference between ROC curves of mean SN_hyper_ volume (*P* = 0.96) on setMag and NM-MRI images

ROC analysis demonstrated that the areas under the curve (AUCs) of mean SN_hyper_ volume for discriminating PD from HCs were 0.904 and 0.906 on setMag and NM-MRI images, respectively. The sensitivity, specificity and accuracy of the mean SN_hyper_ volume on setMag were 83.33, 93.33 and 87.88% with the optimal cutoff value of 84.50 mm^3^, and on NM-MRI images were 88.89, 86.67 and 87.88% with the optimal cutoff value of 88.00 mm^3^, respectively (Table 4). There was no significant difference (*p* = 0.96) between the two ROC curves for differentiating PD from HCs (Fig. 3).

**Table 4:** Receiver operating characteristic analysis of setMag and NM-MRI for the differentiation of PD patients from healthy controls

	AUC	Cut-off value (mm^3^)	Sensitivity (%)	Specificity (%)	Accuracy (%)	*p*
setMag	0.904	≤ 84.50	83.33	93.33	87.88	0.96
NM-MRI	0.906	≤ 88.00	88.89	86.67	87.88	

*P* value is the AUC comparison for mean SN_hyper_ volume to differentiate PD from HCs on setMag and NM-MRI images

### Correlation between mean SN_hyper_ volume and disease severity

There was no significant correlation between the mean SN_hyper_ volume and UPDRSIII score (setMag: *r* = 0.33, *p* = 0.17; NM-MRI: *r* = − 0.07, *p* = 0.78) or H-Y stage (setMag: *r* = 0.24, *p* = 0.35; NM-MRI: *r* = − 0.17, *p* = 0.51).

## Discussion

In this study, we recalculated the setMag images derived from multiple short-echo-time magnitude images of QSM data and found a high spatial similarity (DSC > 0.7) between SN_hyper_ on setMag and SNc on NM-MRI for both HCs and PD patients, whose volumes could differentiate PD from HCs without significant inter-modality difference. Radiological-histological study has confirmed the co-localization of the hyperintensity area on NM-MRI and neuromelanin-containing dopaminergic neurons in SNc [3]. Our results therefore demonstrated that the proposed setMag image could provide neuromelanin contrast by showing substantially similar location of hyperintensity area with SNc on NM-MRI, and comparable PD diagnostic performance with NM-MRI.

The mechanism underlying the contrast of hyperintensity neuromelanin-sensitive area on setMag image may be related with several factors. First, neuromelanin in SNc has specified structural and physical characteristics. Neuromelanin is a macromolecule composed of melanin, proteins, lipids and metal ions [19]. According to in vitro studies in synthetic melanin, the presence of ferric iron in the iron-melanin complex shortened T1 and T2 relaxation times determined by MRI [20, 21]. Thus, although imaging of macromolecules with short T2 is difficult using standard MR sequences [22], specialized sequences utilizing the short T1 and other macromolecule-related characteristics could work. Second, accordingly, the scanning parameters of previous NM-MRI techniques [1, 2], such as repetition time (TR), echo time (TE) and bandwidth, were designed to provide best neuromelanin contrast. The contrasts were mainly based on T1 effects [20] and magnetization transfer (MT) effects [9]. An in vitro study in synthetic melanins [20] confirmed the role of T1 relaxation time reduction in the mechanism underlying contrast on NM-MRI. The first published NM-MRI study in SNc used a T1-weighted fast spin echo sequence (FSE) [1]. As an inherent MT effect was also included in this technique, other studies used explicit MT-MRI sequence and demonstrated the significant contribution of MT effect in neuromelanin-sensitive contrast, in addition to T1-effects [9]. In our study, whilst the NM-MRI were conducted using similar parameters as the previous study [1], the setMag images were generated from the short echo time magnitude images of GRE sequence with a short TR, short TE and small flip angle. Such parameters would produce images with minimal T2* contrast and significant T1 contrast especially between the short T1 neuromelanin in SNc and surrounding tissues. Due to the small flip angle, the contribution of implicit MT effect may be neglectable compared with other MT-based NM-MRI [9]. Thus, the neuromelanin-based contrast on our setMag images may mainly attributed to T1 effect. Finally, we performed an exponential transform in calculation of the setMag images, which nonlinearly strengthened the contrast ratio between neuromelanin-containing area and other tissues.

The volume reduction of SN_hyper_ in PD patients was mainly related with the decreased neuromelanin during the neurodegenerative process. Neuromelanin could play neuroprotective or neurodegenerative roles in dopaminergic neurons [23]. In normal condition, neuromelanin provide neuronal protect by consuming excess cytosolic dopamine and chelating ferric irons [24]. In cases of iron overload such as PD, excess irons not chelated by neuromelanin could cause cell death by oxidative stress. Afterwards, neuromelanin released by dying neurons is phagocytosed and degraded by microglia, results in a decreased amount. Additionally, irons and other toxins previously accumulated by neuromelanin are released, resulting in a self-propelling mechanism of neuroinflammation and neurodegeneration in PD [23]. The significantly reduced volume of hyperintensity area in SNc of PD, as revealed by our results of neuromelanin-sensitive setMag and NM-MRI, could be a representation of this cumulative effect of neuromelanin reduction, and used as a convincible biomarker for exhibiting neuromelanin degeneration in PD.

However, no correlation was found between the mean SN_hyper_ volume and disease severity (UPDRSIII score or H-Y stage) in our results. As previous studies showed controversial results, with weak [25] or no correlation [26–28] with UPDRSIII score or H-Y stage, the volume of SN_hyper_ as a monitoring tool for PD patients could not be determined and needs more evidence.

There are some limitations in the present study. First, as the spatial resolution of setMag and NM-MRI images was not exactly same, we performed co-registration of NM-MRI to setMag images using SPM12 before spatial similarity comparison. The potential misregistration in SN_hyper_ regions may result in an underestimation of the spatial overlap between SN_hyper_ of the two modalities. Although the theoretical similarity between them has been discussed in detail, studies using the exact same spatial resolution could validate this conclusion more intuitively. Second, the SN_hyper_ regions were manually segmented. In PD patients with decreased neuromelanin, the difficulties in delineating this area could be a cause of relatively less DSC value in PD than HC group. Although the segmentation was conducted by two experienced radiologists with high ICC, an automated and objective segmentation method could be beneficial for future studies.

## Conclusion

We proposed a setMag image derived from multiple short-echo-time magnitude images of QSM data, and quantitatively demonstrated that the setMag image could provide neuromelanin contrast by showing substantially similar location with SNc on NM-MRI, and the volume of this hyperintensity area could be used as a promising imaging biomarker for PD diagnosis. Our results show proofs for a new QSM-based multi-contrast imaging strategy, which could provide additional convenience in future PD studies.

## Data Availability

The datasets used and analyzed during the current study are available from the corresponding author on reasonable request.

## Abbreviations

SN: Substantia nigra
SNc: Substantia nigra pars compacta
SN_hyper_: Hyper intensity areas of substantia nigra
NM-MRI: Neuromelanin-sensitive MRI
QSM: Quantitative susceptibility mapping
setMag: Short echo time magnitude image
PD: Parkinson’s disease
HCs: Healthy controls
